# Betanidin significantly reduces blood glucose levels in BALB/c mice fed with an atherogenic diet

**DOI:** 10.1007/s13659-012-0034-z

**Published:** 2012-06-22

**Authors:** Agustin Lugo-Radillo, Ivan Delgado-Enciso, Elpidio Peña-Beltrán

**Affiliations:** 1 Instituto Nacional de Geriatría, Periférico Sur 2767, San Jerónimo Lídice, Magdalena Contreras, 10200 México D.F., México; 2 Facultad de Medicina, Universidad de Colima, Av. Universidad 333, Colonia Las Víboras, 28040 Colima, Colima, México; 3 Facultad de Ciencias Biológicas y Agropecuarias, Universidad de Colima., Carr. Colima-Manzanillo km 40, 28100 Tecomán, Colima, México

**Keywords:** betalains, betanidin, blood glucose, hypoglycemiant

## Abstract

Six weeks BALB/c mice were fed with an atherogenic diet for 24 weeks and purified water *ad libitum.* An experimental group was given betanidin, orally, during the last 40 days of the experiment at a dose of 9.6 mg per mouse per day. Negative controls were fed with standard rodent chow only. Glycemia was measured at the end of the experiment, after overnight fasting. The group treated with betanidin presented a highly significant reduction of 50.94% compared to positive controls. We conclude that betanidin reduces glycemia in BALB/c mice by an unidentified mechanism.

**Electronic Supplementary Material:**

Supplementary material is available for this article at 10.1007/s13659-012-0034-z and is accessible for authorized users.

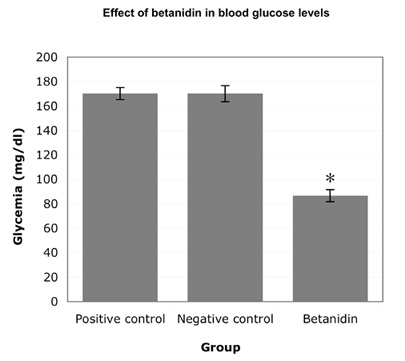

## Electronic supplementary material

Supplementary material, approximately 153 KB.
