# Supplementary material for: Betanidin significantly reduces blood glucose levels in BALB/c mice fed with an atherogenic diet
Source: Nat Prod Bioprospect. 2012 Jun 22;2(4):154–5. doi: 10.1007/s13659-012-0034-z (PMC4131630; doi:10.1007/s13659-012-0034-z)
Supplement: Supplementary file 1 — Supplementary material, approximately 153 KB. [file 13659_2012_34_MOESM1_ESM.pdf]

Electronic Supplementary Material

Betanidin significantly reduces blood glucose levels in BALB/c mice fed with an atherogenic diet

Agustin LUGO-RADILLO,[a,b,]* Ivan DELGADO-ENCISO,[b] and Elpidio PEÑA-BELTRÁN[c]

[a]Instituto Nacional de Geriatría, Periférico Sur 2767, San Jerónimo Lídice, Magdalena Contreras, 10200 México D.F., México

[b]Facultad de Medicina, Universidad de Colima, Av. Universidad 333, Colonia Las Víboras, 28040 Colima, Colima, México

[c]Facultad de Ciencias Biológicas y Agropecuarias, Universidad de Colima. Carr. Colima-Manzanillo km 40. 28100 Tecomán, Colima, México

Received 9 May 2012; Accepted 15 June 2012

Abstract: Six weeks BALB/c mice were fed with an atherogenic diet for 24 weeks and purified water *ad libitum*. An experimental group was given betanidin, orally, during the last 40 days of the experiment at a dose of 9.6 mg per mouse per day. Negative controls were fed with standard rodent chow only. Glycemia was measured at the end of the experiment, after overnight fasting. The group treated with betanidin presented a highly significant reduction of 50.94% compared to positive controls. We conclude that betanidin reduces glycemia in BALB/c mice by an unidentified mechanism.

*To whom correspondence should be addressed. E-mail: alugor@hotmail.com

Plant Material and Sample Preparation. Fresh red-purple pitaya fruits (*Hylocereus ocamponis*) was purchased from a comercial plantation in Jalisco (Mexico). Upon arrival of fruits, they were washed and peeled by hand. Skins were discarded and the fruit flesh was macerated in a blender until being completely homogenized. For pigment extraction, 1 part of this sample was shaken with 2 parts of solvent for 5 minutes and allowed to stand for 15 min; solvents used were 80% acetone, 80% methanol and ultra purified water. After, the mixture was filtrated through a nylon cloth and centrifuged at 3500 rpm for 15 min. Afterwards, the supernatant was filtered through YM-10 membranes (Millipore) to remove proteins, and the filtrate was used for pigment analysis and further purification. All was performed at room temperature.

FPLC Purification. Anionic exchange chromatography of betanin from pitaya was performed in an Äkta purifier apparatus (Amersham Biosciences). The equipment was operated via a PC using Unicorn software version 5.20. Elutions were followed at 280 and 536 nm. Solvents used were sodium acetate buffer 10 mM, pH 5.0 (solvent A) and sodium acetate buffer 10 mM, pH 5.0, with NaCl 2 M (solvent B). A 25 × 7 mm, 1 mL Q-Sepharose Fast Flow column (cross-linked agarose with quaternary ammonium as exchanger group, 90 μm of particle size) (Amersham Biosciences) was used. After sample injection, the elution process was as follows: 0% B from beginning to 7 mL; after washing, a linear gradient was developed from 0% B to 35% B in 20 mL, with 1 mL fractions being collected. Cleaning (7 mL, 50% B) and re-equilibration (7 mL, 100% A) steps were performed between each sample injection. Injection volume was 1 mL, and the flow rate was 0.5 mL min-1. A total of 100 μL was injected for analytical purposes and comparison with standards.

HPLC Analysis. Varian ProStar apparatus equipped with a UV-VIS Varian 9050 detector (Varian, USA) was used for analytical HPLC. Reversed phase chromatography was performed with a 250 × 4.6 mm Varian C-18 column packed with 5 μm particles (Varian, USA). Gradients were formed between two helium degassed solvents. Solvent A was water acidified with 0.05% trifluoroacetic acid, and solvent B was composed of acetonitrile with 0.05% trifluo- roacetic acid. Linear gradient was performed in 20 min from 0% B to 28% B.

Betanidin Obtainment. After, betanidin was obtained enzymatically from purified betanin through β-glucosidase (EC 3.2.1.21, β-D-glucoside glucohydrolase) treatment. A 4 μM betanin solution was incubated for 30 min with 14 units mL-1 of β-glucosidase in 50 mM sodium acetate buffer, pH 5.0, at 25 °C. The enzyme was removed by ultrafiltration through YM-10 membranes. Transformation

was complete according to HPLC analysis.

Absorbance Spectroscopy. For absorbance spectroscopy, a Uvikon 940 spectrophotometer (Kontron Instruments, Zurich, Switzerland) was used. Consecutive wavelength scans were performed in a BioPhotometer plus spectrophotometer diode array UV-visible spectrophotometer (Eppendorf, Germany).

Quantification of Betanin and Betanidin. Betanin and betanidin concentrations were evaluated through absorbance, taking a molar extinction coefficient of ε) 65 000 M-1 cm-1 and ε) 54 000 M-1 cm-1, respectively, at 536 nm. Measurements were made in water at 25 °C.

Glycemia Results.

Glucose mg/dl	
177	Negative control
186	Negative control
162	Negative control
163	Negative control
121	Negative control
157	Negative control
171	Negative control
161	Negative control
175	Negative control
223	Negative control
112	Negative control
181	Negative control
196	Negative control
183	Negative control
176	Negative control
178	Negative control

Average	170.125
SEM	6.561805519

Glucose mg/dl	
198	Positive control
157	Positive control
145	Positive control
189	Positive control

175	Positive control
167	Positive control
158	Positive control
144	Positive control
182	Positive control
178	Positive control
184	Positive control
184	Positive control
151	Positive control

Average	170.1538462
SEM	4.905316522

Glucose mg/dl	
102	Betanidin
84	Betanidin
105	Betanidin
92	Betanidin
83	Betanidin
95	Betanidin
108	Betanidin
76	Betanidin
64	Betanidin
105	Betanidin
70	Betanidin
56	Betanidin

Average	86.66666667
SEM	5.020161372
